# Tumor expression of survivin, p53, cyclin D1, osteopontin and fibronectin in predicting the response to neo-adjuvant chemotherapy in children with advanced malignant peripheral nerve sheath tumor

**DOI:** 10.1007/s00432-018-2580-1

**Published:** 2018-01-13

**Authors:** Gabrielle Karpinsky, Malgorzata A. Krawczyk, Ewa Izycka-Swieszewska, Aleksandra Fatyga, Agnieszka Budka, Walentyna Balwierz, Grazyna Sobol, Beata Zalewska-Szewczyk, Magdalena Rychlowska-Pruszynska, Teresa Klepacka, Bozenna Dembowska-Baginska, Bernarda Kazanowska, Anna Gabrych, Ewa Bien

**Affiliations:** 1 Children’s Hospital of Michigan, 3901 Beaubien St, Detroit, MI USA; 2 Department of Pediatrics, Hematology and Oncology, Medical University of Gdansk, 7 Debinki Street, 80-211 Gdansk, Poland; 3 Department of Pathology and Neuropathology, Medical University of Gdansk, 1 Debinki Street, Gdansk, Poland; 4 Department of Pediatrics, Hematology and Oncology, University Clinical Centre, 7 Debinki Street, Gdansk, Poland; 5 Department of Pediatric Oncology and Hematology, Jagiellonian University Medical College, 265 Wielicka Street, Krakow, Poland; 6 Department of Pediatrics, Medical University of Silesia, 15 Medykow Street, Katowice, Poland; 7 Department of Pediatrics, Oncology, Hematology and Diabetology, Medical University of Lodz, 36/50 Sporna Street, Lodz, Poland; 8 Department of Surgical Oncology, Institute of Mother and Child, 17A Kasprzaka Street, Warsaw, Poland; 9 Department of Pathology, Institute of Mother and Child, 17A Kasprzaka Street, Warsaw, Poland; 10 Department of Oncology, Children’s Memorial Health Institute, 20 Dzieci Polskich Street, Warsaw, Poland; 11 Department of Pediatric Bone Marrow Transplantation, Oncology and Hematology, Wroclaw Medical University, 213 Borowska Street, Wroclaw, Poland

**Keywords:** Survivin, P53, Cyclin D1, Osteopontin, Response to neo-adjuvant chemotherapy, Malignant peripheral nerve sheath tumor, Children

## Abstract

**Purpose:**

Selected cell-cycle regulators and extracellular matrix proteins were found to play roles in malignant peripheral nerve sheath tumor (MPNST) biology. We aimed to analyze whether initial tumor tissue expressions of survivin, p53, cyclin D1, osteopontin (OPN) and fibronectin (FN) correlate with the response to neo-adjuvant CHT (naCHT) in children with advanced inoperable MPNST.

**Methods:**

The study included 26 children with MPNST (*M*/*F* 14/12, median age 130 months) treated in Polish centers of pediatric oncology between 1992 and 2013. Tissue expression of markers was studied immunohistochemically in the manually performed tissue microarrays and assessed semi-quantitatively as low and high, based on the rate of positive cells and staining intensity.

**Results:**

Good response to naCHT was noted in 47.6%, while poor—in 52.4% of patients. The response to naCHT was influenced negatively by the presence of neurofibromatosis NF1 and high initial tumor tissue expression of OPN, survivin, p53 and cyclin D1. Patients with high tumor expression of either OPN, survivin or p53 and those with simultaneous high expression of ≥ 3 of the markers, responded significantly worse to naCHT, than patients, in whom expression of ≤ 2 markers were detected at diagnosis. Nearly, 85% of patients expressing ≥ 3 markers, responded poor to CHT; while 87.5% of children, expressing ≤ 2 markers, were good responders.

**Conclusion:**

The initial tumor tissue expression of OPN, survivin, p53 and cyclin D1 may serve as markers to predict response to naCHT in pediatric advanced MPNST. Future studies in more numerous group of patients are needed to confirm these preliminary results.

## Introduction

Malignant peripheral nerve sheath tumors (MPNST) are rare soft tissue sarcomas (STS) of neurogenic origin. Most cases arise in adulthood, however, 10–20% of MPNST occur in the first decades of life. MPNST may arise from pre-existing neurofibromas, particularly in patients with neurofibromatosis type 1 (NF1), or de novo (Ducatman et al. 1986). MPNST typically localize in the trunk, extremities and head and neck, with the majority of lesions being deep seated. The mainstay of treatment of MPNST, both in children and adults, is complete surgical resection with negative margins.

However, most cases of MPNST are diagnosed in advanced stages with invasive and/or metastatic tumors, making complete surgery infeasible as a primary procedure. Postoperative radiotherapy (RTX) is recommended for local control in patients in whom incomplete resection was performed. Nevertheless, the prognosis in such patients remains unsatisfactory—with the 5-year-overall survival (5-y-OS) rate of approximately 34–44% for children and adults (Ducatman et al. 1986).

The effectiveness of neo-adjuvant CHT (naCHT) in patients with initially inoperable MPNST is uncertain. Some authors have regarded MPNST as a chemoresistant malignancy (Zehou et al. 2013; Kolberg et al. 2013). However, in pediatric MPNST, the post-CHT tumor regression was shown to enable complete delayed resections in a proportion of patients, which, in turn, improved the outcome (Carli et al. 2005; Ferrari et al. 2011). This therapeutic strategy has been accepted and used in most protocols recommended for pediatric MPNST (Treuner et al.^1991^). Most authors report that the response to CHT is significantly worse in patients with NF1 (Ferrari et al. 2011). Additionally, the chemosensitivity of MPNST cannot be predicted based on clinical features of the disease in particular patients. Therefore, it is important to define new markers reflecting the biological aggressiveness of MPNST, which might help to optimally select candidates most likely to benefit from intense naCHT.

Recent studies suggest that MPNST growth is a multistage process that may involve a number of altered cell-cycle regulators. Among them, survivin, p53, and cyclin D1 have been associated with the deregulation of the cellular proliferation and worse OS in sarcomas (Zhou et al. 2003). Moreover, the extracellular matrix (ECM) glycoproteins, such as: osteopontin (OPN) and fibronectin (FN), have been shown to facilitate tumor progression and metastasis of malignancies, including sarcomas (Ioachim et al. 2002; Weber et al. 2010).

Cell-cycle checkpoint regulator, survivin, is a protein minimally expressed in healthy tissues. As a member of the inhibitor of apoptosis protein (IAP) family, survivin regulates cell division at G2 phase. Its accumulation interferes with the activation of caspase activity during programmed cell death and thus promotes cell growth (Brady et al. 2015). High expression of survivin has been correlated with poor clinical outcomes in various carcinomas, neuroblastoma (Ito et al. 2005), medulloblastoma (Fangusaro et al. 2005) and also with the chemoresistance in adult rhabdomyosarcoma (RMS) and MPNST (Caldas et al. 2006).

*TP53* is a tumor suppressor gene, located on chromosome 17, responsible for modulating the function of proteins that protect cells from apoptosis in response to stress (Partridge et al. 2007). It has been recently stated that *TP53* mutations are present in 75% of MPNST tumors (Kindblom et al. 1995). Overexpression of p53 protein and mRNA were found in MPNST, as compared to benign neurofibromas (Leroy et al. 2001). In addition, p53 reactivity was more frequent in NF1-associated MPNST (Zhou et al. 2003). These findings may indicate that a p53-mediated pathway plays a role in the development and growth of MPNST (Cunha et al. 2012). A close relation between the regulation of p53 and survivin, reported in many adult carcinomas, supports the involvement of these markers in the processes of tumor cell apoptosis and survival (Sarela et al. 2002).

Cyclin D1 is a proto-oncogene, responsible for the activation of cyclin-dependent kinases which proceed to phosphorylate retinoblastoma (Rb) protein and activate transcription factors involved in the transition from G1 to S phase in the cell cycle (Cordon-Cardo et al. 1995). The overexpression and amplification of cyclin D1 contributes to tumor genesis and has been associated with poor prognosis of many cancers of adults, including: esophageal, colon, prostate, pancreas, and bladder cancers (Ikeguchi et al. 2001; Gansauge et al. 1997). The overexpression of cyclin D1 has been associated with worse OS and higher tumor grade in STS of the extremities and retroperitoneum (Kim et al. 1998).

OPN and FN are important components of ECM, facilitating the growth of a variety of human cancers (Ioachim et al. 2002; Weber et al. 2010). Both markers have been involved in the processes of cell adhesion and migration, blood coagulation, host defenses, tumor invasion and early metastases. In cancer, OPN plays multifaceted roles in promoting the tumor progression by regulating cell–matrix interactions and cellular signaling, inhibiting apoptosis of tumor cells (Standal et al. 2004) and stimulating neo-angiogenesis (Anborgh et al. 2011). OPN overexpression has been linked to an unfavorable prognosis in many adult malignancies, including sarcomas (Bramwell et al. 2005). FN is produced by various types of benign and malignant epithelial and mesenchymal cells, being involved in a number of processes crucial to the tumors’ invasion and metastasis (Knowles et al. 2013). FN is an inducer and one of the markers of the cells’ mesenchymal phenotype, which is upregulated during the epithelial–mesenchymal transition (EMT), through which the epithelial tumor cells achieve more aggressive phenotype and invasive properties. Several studies confirm the role of FN in the pathogenesis, invasiveness and lung metastases formation of adult RMS (Ito et al. 2004).

The overexpression of survivin, p53, cyclin D1, OPN and FN may reflect the inner biology of MPNST, with respect to cell proliferation, invasion and escape from apoptosis. In the present study, we investigated the role of tumor expression of survivin, p53, cyclin D1, OPN, and FN in the prediction of the response to naCHT in children with advanced MPNST.

## Materials and methods

### Patients’ characteristics

The study included 26 children with MPNST (14M/12F; age range, 2–252 months; median age, 130 months), registered in the Polish Pediatric STS Registry. All MPNST diagnoses were performed by pathologists from two independent institutions. For the purpose of this study, the tumor specimens were reviewed again by one of the authors (E.I-S.). The stages of MPNST were defined according to both clinical tumor nodes metastases (TNM) pre-treatment staging system and the Intergroup Rhabdomyosarcoma Study (IRS) post-surgical grouping system.

All children were treated between III’1992 and XI’2013, using a multi-modality therapeutic CWS (Cooperative Weichteilsarkom Study Group) protocols, including surgery, CHT, and RTX. The surgical tumor resection performed as an initial measure was termed primary excision (PE). Microscopically complete surgery was termed R0 (IRS I), microscopically incomplete—R1 (IRS II), excision with macroscopic residues and biopsy only—R2 (IRS III). IRS IV meant metastatic disease at diagnosis. RTX with the use of external beam irradiation was administered to patients considered at risk of local recurrence. Chemotherapeutic schemes did not change substantially over the years and comprised cycles composed mostly of: vincristine, ifosfamide, dactinomycin, anthracyclines (doxorubicin or epirubicin), etoposide and carboplatin. The combination of drugs depended on the risk group classification and response to initial CHT.

Response to naCHT was evaluated after three courses of CHT (between 9th and 10th weeks of treatment). It was based on the reduction in volume of all measurable lesions, using the following criteria: complete response (CR) = complete disappearance of disease; good response (GR)—tumor reduction ≥ 67% <100%; partial response (PR) = tumor reduction ≥ 33 < 67%; stable disease (SD) = tumor reduction < 33%; progression of disease (PD) = increase in tumor size or new lesion detection. “Good response” to CHT comprised CR, GR and PR, while SD and PD were termed “poor response” to CHT.

### Immunohistochemical analysis of markers

The study was carried out in 26 archival histological samples of fresh-frozen paraffin-embedded (FFPE) primary, chemo-naive MPNST tumors, obtained at diagnosis of pediatric patients diagnosed and treated in Polish oncologic centers between 1992 and 2013. The samples were marked in a way preventing identification by other persons.

Pathological analyses and IHC testing were performed in the Department of Pathology and Neuropathology Medical University of Gdansk (MUG). From the representative sections, the tissue microarrays (TMA) was constructed, using the commercially available Tissue -Tek® Quick-Ray ™ TMA System kit, of the Sakura Finetek USA, Inc. The IHC stainings were performed on TMA, using monoclonal antibodies to detect analyzed markers. The appropriate positive and negative controls were introduced. The qualitative and quantitative analysis of protein expression in MPNST tissue was performed using the digital image analyzer. The immunophenotyping was made by the visualization system Dako, Denmark.

**Fig. 1:**
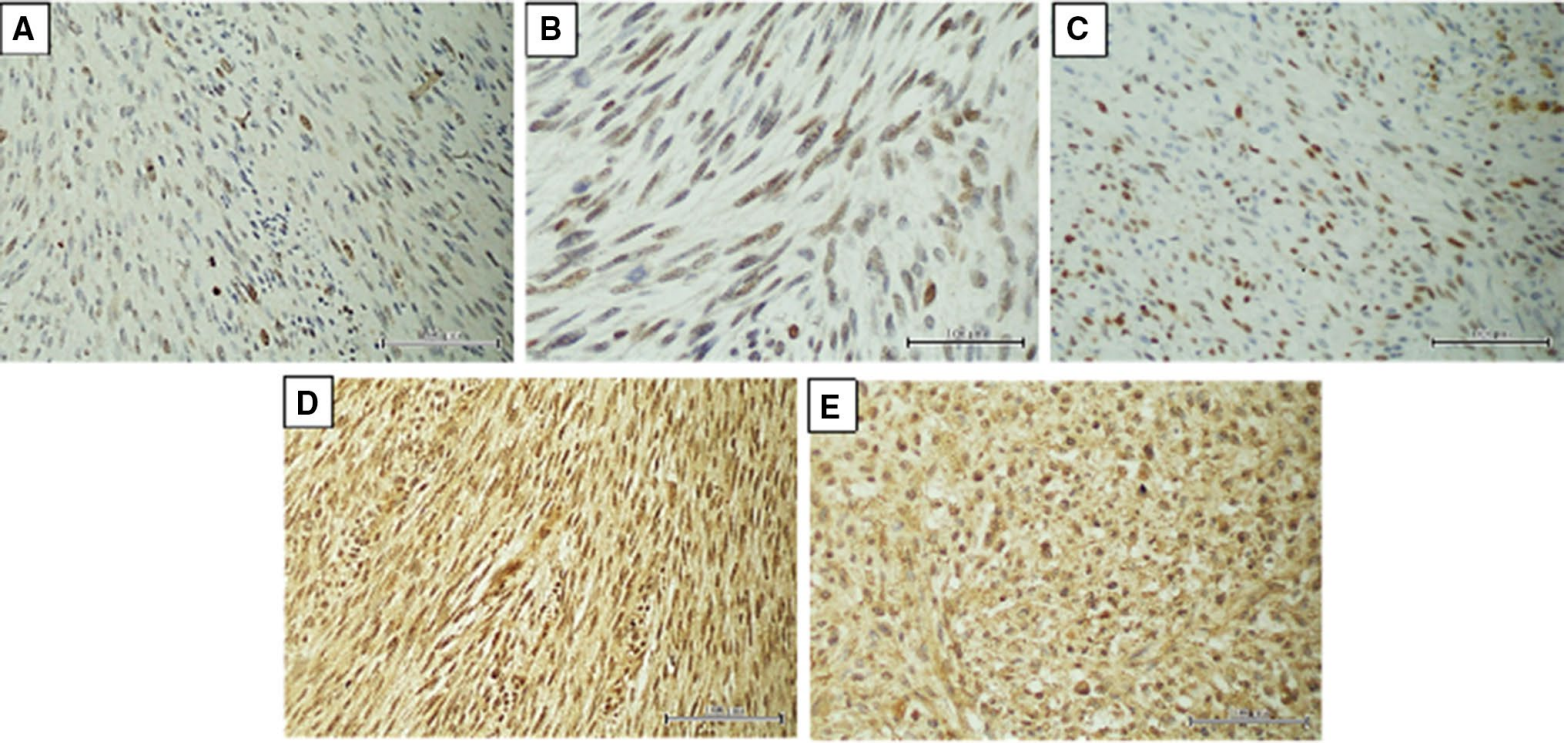
The immunostaining of the analyzed markers in MPNST samples. **a** Low expression of survivin (×200). **b** The p53 strong immunoreactivity (×400). **c** High cyclin D nuclear staining (strong immunoreactivity in more than 50% of cells). **d** High diffuse nuclear and cytoplasmatic immunoreactivity of osteopontin (×200). **e** Fibronectin high expression in more than 90% cells (×200)

The expressions of analyzed markers in FFPE tumors were assessed using a semiquantitative method, based on the intensity of color reaction (on a scale of + to +++), and the rate of immunopositive cells (within the ranges of 0–5, 6–25, 26–50, > 50%). Based on these two variables, a numerical ratio of the markers’ expression was estimated and used for further analyses. Its value determined the expression of markers as low or high. Figure 1a–e shows the immunostainings of the analyzed markers in MPNST samples.

The study was approved by the Independent Bioethical Committee of the MUG, Poland (No. NKBBN/449/2013).

### Statistical analysis

The results were subjected to statistical study, using computer statistical software package EPIINFO Ver. 7.1.1.14 (02-07-2013). *p* < 0.05 was considered statistically significant.

## Results

The clinical characteristics of the study group and the results of the tumor expressions of analyzed markers are presented in Table 1.

**Table 1:** Clinical characteristics of the study group and the results of IHC markers’ expressions in children with MPNST

Case	NF1	Max tumor size (mm)	Location	TNM	PE	IRS Stage	Survivin	p53	Cyclin D1	OPN	FN	Response to CHT	Outcome
1	No	250	Unfavorable–deep	T2N1M0	R1	II	1	1	1	1	1	Poor (PD)	DOD
2	Yes	100	Unfavorable–superficial	T2N1M0	R2	III	1	0	0	1	0	Good (PR)	Alive
3	No	51	Unfavorable–deep	T2N1M1	Bx	IV	1	1	0	1	1	Poor (SD)	DOD
4	No	155	Unfavorable–superficial	T2N0M0	Bx	III	1	1	0	0	1	Poor (SD)	Alive
5	No	55	Unfavorable–deep	T2N0M0	R1	II	0	0	0	0	0	N/A	Alive
6	No	80	Unfavorable–superficial	T2N0M1	R2	IV	1	0	0	1	0	Good (PR)	Alive
7	No	100	Unfavorable–deep	T2N0M0	R2	III	0	1	0	0	1	Good (GR)	Alive
8	Yes	86	Unfavorable–superficial	T2N0M0	Bx	III	1	1	1	1	0	Good (PR)	DOD
9	Yes	235	Unfavorable–deep	T2N0M0	Bx	III	1	0	1	1	1	Poor (PD)	DOD
10	No	25	Unfavorable–deep	T2N0M0	R1	II	0	0	1	1	1	N/A	DOD
11	Yes	191	Unfavorable–deep	T2N0M1	Bx	IV	1	1	1	1	1	Poor (PD)	DOD
12	Yes	300	Unfavorable–deep	T2N1M1	Bx	IV	0	1	1	1	1	N/A—died of CHT complications	DOD
13	Yes	29	Favorable–deep	T2N0M0	R2	III	1	1	1	1	0	Poor (PD)	DOD
14	No	40	Unfavorable–deep	T2N0M0	Bx	III	1	0	0	0	0	Poor (SD)	Alive
15	Yes	145	Unfavorable–deep	T2N1M0	R2	III	1	0	1	1	0	Good (PR)	DOD
16	No	100	Unfavorable–superficial	T2N0M0	Bx	III	0	0	0	1	0	Good (PR)	Alive
17	No	150	Unfavorable–superficial	T2N0M0	R1	II	1	0	0	0	1	N/A	Alive
18	No	55	Unfavorable–deep	T2N0M0	R2	III	0	0	0	0	1	Good (CR)	Alive
19	No	48	Unfavorable–deep	T2N0M0	Bx	III	0	0	0	0	1	Good (PR)	Alive
20	Yes	65	Unfavorable–deep	T2N0M0	Bx	III	1	1	1	1	1	Poor (SD)	Alive
21	No	30	Favorable–deep	T2N0M0	Bx	III	1	1	1	1	1	Poor (PD)	Alive (surg)
22	Yes	53	Unfavorable–deep	T2N0M0	Bx	III	1	1	1	1	1	Poor (SD)	DOD
23	No	90	Unfavorable–deep	T2N0M1	Bx	IV	1	1	1	1	1	Poor (SD)	DOD
24	No	43	Unfavorable–superficial	T1N0M0	R2	III	1	0	1	0	1	Good (PR)	Alive
25	No	69	Unfavorable–superficial	T1N0M0	Bx	III	0	0	0	0	0	Good (PR)	Alive
26	Yes	30	Unfavorable–superficial	T1N0M0	R1	II	1	1	0	0	0	N/A	Alive

*NF1* neurofibromatosis type 1, *T1* noninvasive tumor, *T2* invasive tumor, *N0* no lymph node involvement, *N1* lymph node involvement, *M0* no distant metastases, *M1* distant metastases, *Bx* biopsy, *R0* microscopically complete resection, *R1 (IRS II)* microscopically incomplete resection, *R2 (IRS III)* macroscopically incomplete resection, *PE* primary excision; *CHT* chemotherapy; *RT* radiotherapy; unfavorable tumor sites: head and neck parameningeal region, bladder and prostate, inner organs, retroperitoneum, extremities, perineum and other; favorable tumor sites: head and neck non-parameningeal region, urogenital tract without bladder, prostate, orbit; N/A: not applicable (no measurable tumor present); *CR* complete response; *GR* good response; *PR* partial response; *SD* stable disease; *PD* progression of disease; *DOD* died of disease

Over 80% (21/26) of patients were diagnosed in highly advanced stages of disease—inoperable (*n* = 16; IRS III) or metastatic (*n* = 5; IRS IV). The primary tumors were located in unfavorable sites, including: head and neck parameningeal region, bladder and prostate, inner organs, retroperitoneum, extremities and other in 24 (92%) patients. Most of the tumors were large (> 5 cm in 20 children, 77%; > 10 cm in 10 children, 38%), invasive (T2 feature) in 88% of patients and deeply seated (17 patients, 65%). In ten patients (38%), NF1 was diagnosed. The 5-year-event free survival (EFS) for the whole study group was 35.6 ± 9.7% and 5-y-OS—62.2 ± 10.2%. Median EFS was 19.8 months and median OS was 51 months.

Response to naCHT was assessable in 21 children (81%). Five patients, who were not assessable, included: four children with no macroscopic tumor residue after PE and one child after R2 PE, who died of complications soon after treatment introduction. Ten patients (47.6%) achieved good response to naCHT (CR in one, GR in one and PR in eight), while 11 patients (52.4%)—poor response to naCHT (SD in six and PD in five). The response to naCHT and prognosis were significantly worse in NF1 patients. Among ten patients with NF1, only three achieved good response to naCHT. Within 16 patients without NF1 good response to naCHT was stated in 7 children (43.8%). All of them are alive and without evidence of disease (Table 2).

**Table 2:** NF1 correlation to CHT response and outcome in selected patients

	NF 1 (+)			NF 1 (−)		
	Alive	Dead	*n*	Alive	Dead	*n*
Good response (CR + GR + PR)	1	2	3	7	0	7
Poor response (SD + PD)	1	4	5	4	2	6
N/A	1	1	2	2	1	3
Total	3	7	10	13	3	16

*NF1* neurofibromatosis type 1; *CR* complete response; *GR* good response; *PR* partial response; *SD* stable disease; *PD* progression of disease; *DOD* died of disease; *N/A* no data; *n* patients number; (+), positive; (−), negative

### Markers analysis

The results of the IHC expressions of survivin, p53, cyclin D1, OPN, and FN in particular patients together with their clinical characteristics and outcome are displayed in Table 1. The comparison between the expressions of analyzed markers in good and poor responders are shown in Table 3.

**Table 3:** The tumor expression of analyzed markers in poor and good responders with MPNST

High markers’ expression in tumor	*n*	Good response to CHT	Poor response to CHT	*p*
P53 (+)	11	2	9	0.00892F
P53 (−)	10	8	2	
OPN (+)	14	5	9	0.0183F
OPN (−)	7	5	2	
Survivin (+)	16	5	11	0.0124F
Survivin (−)	5	5	0	
FN (+)	13	4	9	0.0805F
FN (−)	8	6	2	
Cyclin D1 (+)	11	3	8	0.0861F
Cyclin D1 (−)	10	7	3	
Survivin (+) and cyclin D1 (+)	11	3	8	0.0256F
Both (−)	5	5	0	
p53 (+) and cyclin D1 (+)	8	1	7	0.0101
Both (−)	7	6	1	
OPN (+) and cyclin D1 (+)	10	2	8	0.118F
Both (−)	6	4	2	
Survivin (+) and OPN (+)	13	4	9	–
Both (−)	4	4	0	
Survivin (+) and p53 (+)	10	1	9	–
Both (−)	4	4	0	
Survivin (+) and FN (+)	10	1	9	–
Both (−)	2	2	0	
OPN (+) and p53 (+)	9	1	8	0.0230
Both (−)	5	4	1	
OPN (+) and FN (+)	8	0	8	–
Both (−)	2	1	1	
OPN (+) and p53 (+) and cyclin D1 (+)	9	1	8	0.00548
Both (−)	10	8	2	
Survivin (+) and OPN (+) and p53 (+)	9	1	8	0.00874
Maximum 1 marker (+)	7	6	1	
OPN (+) and survivin (+) and cyclin D1 (+)	10	2	8	0.0584F
Maximum 1 marker (+)	7	5	2	
OPN and survivin and p53 and cyclin D1—at least 3 of 4 markers (+)	11	2	9	0.00892F
Maximum 2 markers (+)	10	8	2	

*OPN* osteopontin, *FN* fibronectin, *CHT* chemotherapy, *F* Fisher’s test, (+) high immunostaining of the tumor tissue, (−) low immunostaining of the tumor tissue

### The expression of particular markers in relation to the response to CHT

Survivin was expressed in 16/21 (76%) patients, OPN in 14 (67%), FN in 13 (62%), cyclin D1 in 11 (52%) and p53 in 11 (52%). Figure 1a–e shows various immunoreactivities of the analyzed markers in MPNST tumors. Additionally, significant positive correlations were found between cyclin D1 and p53 (*p* = 0.010), OPN and p53 (*p* = 0.023) and cyclin D1 and FN (*p* = 0.032).

Patients with poor response to naCHT demonstrated high expressions of analyzed markers more frequently than patients with good response to naCHT. In poor responders, survivin was expressed in all patients, OPN in 81.8%, cyclin D1 in 72.7%, p53 in 81.8%, and FN in 81.8%. In good responders, the expression of analyzed markers was less frequent (50, 20, 50, 40 and 20%, respectively). All patients expressing negatively for survivin (*n* = 5) responded well to naCHT (CR-1, GR-1, PR-3). In the univariate analysis, it was shown that high expressions of survivin, p53, and OPN were significantly associated with a poor response to naCHT (*p* = 0.009, *p* = 0.012 and *p* = 0.018, respectively). The tumor expressions of cyclin D1 and of FN were found higher in poor responders, than in good responders, however, the differences between the groups were insignificant (*p* = 0.086, *p* = 0.080, respectively).

### The number of markers expressed simultaneously in relation to response to naCHT

Only one patient did not display any marker’s expression. This patient achieved PR to naCHT and was alive at the last observation, 16 months after therapy discontinuation. The expression of one marker was found in four patients, two markers—in three patients, three markers—in three, four markers—in four and all five markers in six patients. Among four children, who expressed only one marker (patients 14, 16, 18 and 19), two achieved PR, one CR and one SD. All these children survived. The only patient in our study to achieve CR was found to express FN alone.

Among 13 patients, who expressed three or more markers, 11 children (84.6%) responded poor to naCHT and only two (15.4%) achieved PR to naCHT. To the contrary, among eight children expressing no more than two markers, there were seven good responders (87.5%) and only one poor responder (SD, 12.5%). Six children, who expressed all five analyzed markers, had poor response to naCHT. To the contrary, four of five patients who did not express any of the markers or expressed only one marker, responded well to naCHT.

It was observed that simultaneous high expression of particular markers in one tumor sample was associated with worse response to naCHT (Table 3). For example, the majority of patients expressing positively for both survivin and cyclin D1 (8/11, 73%) responded poor to naCHT, while five patients with no expression of these two markers responded well. The difference between these two groups reached statistical significance (*p* = 0.026). Similarly, poor response to CHT was significantly more frequent in children with MPNST with simultaneous high expressions of p53 and cyclin D1, and of OPN and p53, in comparison to patients not expressing any of these markers (*p* = 0.010; *p* = 0.023; respectively). Due to low number of cases, it was impossible to assess the statistical difference between subgroups of patients with and without high expressions of survivin and OPN, survivin and p53, survivin and FN, and OPN and FN. However, in all subgroups of patients with high expressions of these pairs of markers, poor response to naCHT predominated. It was noted that simultaneous high expressions of OPN, p53 and cyclin D1 was significantly more common in poor responders than no expression of the markers (*p* = 0.005). In addition, high simultaneous expressions of survivin, OPN and p53 were associated with significantly worse response to naCHT, than high expression of a maximum one of these markers (*p* = 0.009). Patients, in whom high tumor tissue immunoreactivity towards OPN, survivin, p53 and cyclin D1, or just three of the four markers were stated at diagnosis, responded significantly worse to CHT than patients, who expressed no more than two of these markers (*p* = 0.009).

## Discussion

The aim of the study was to assess whether selected proteins regulating the cell-cycle, tumor cells apoptosis and tumor invasion, i.e.: survivin, p53, cyclin D1, OPN and FN, may become potential biomarkers to predict the response to naCHT in advanced pediatric MPNST. Identification of such biomarkers would allow for more individualized treatment based on optimal selection of candidates for multidrug naCHT. This would diminish the risk of unnecessary CHT-related complications in those, who are not likely to benefit from CHT.

It is indisputable that complete surgical tumor excision with negative (wide) margins offers the best outcome, both in children and adults (Carli et al. 2005; Ferrari et al. 2011). However, in the majority of children with MPNST, the disease is diagnosed late, in the phase of invasive, inoperable and/or metastatic tumor, which is not feasible for the upfront complete resection. In such cases, the CWS protocols recommend the naCHT, aimed to shrink the tumor preoperatively. The chemotherapeutic courses administered to children with stage III and IV MPNST, reported in our study, did not change substantially over the years of analysis. In patients with MPNST stage III, most commonly VAIA III regimen was administered, including the alternating courses, containing ifosfamide, vincristine, dactinomycin (I2VA) or ifosfamide, vincristine and doxorubicin (I2VAdr). Children with stage IV MPNST received the CEVAIE regimen, composed of I3VAdr, (ifosfamide, vincristine and doxorubicin), CEV (carboplatin, vincristine and epirubicin) and I3VE (ifosfamide, vincristine and etoposide) courses.

The rate of good responses to naCHT among 21 evaluable patients, was 48%. This was similar to the results of Carli et al., who reported responses to CHT in 45% of children (30 out of 64 evaluable). However, in their study, much better outcome was noted in patients treated with naCHT-containing ifosfamide (VAIA, IVA, CEVAIE, 65%), than in those receiving regimens including cyclophosphamide (VACA, VAC/CAV, 17%) or cisplatin and etoposide (20%) (Carli et al. 2005). In our series, all patients with stage III and IV received naCHT-containing ifosfamide. The cumulative dose of ifosfamide during the first-line treatment of both stage III and stage IV MPNST was 54 g/m2.

In clinical practice, it is difficult to predict, who will benefit from naCHT, and in whom this treatment is of no value. Analysis of clinical markers, such as: tumor size, location, invasiveness, lymph node involvement and presence of metastases, does not help to predict the chemosensitivity of MPNST. However, most literature data report on the worse response to naCHT in NF1-related MPNST, as compared to sporadic cases (Ferrari et al. 2011). In the Italian and German series of 167 consecutive pediatric patients with MPNST, recruited over a 25-year period, the response to naCHT was found 18% NF-1 positive and 55% in NF1(-) patients (Carli et al. 2005). Similarly, in our material, the response to naCHT and survival rates were significantly negatively influenced by the presence of NF1. Additionally, in our series, the rate of NF1(+) patients was higher than in the study reported by Cari et al. (38% vs. 11%), which might have caused worse response to naCHT, even if all patients received ifosfamide-containing course.

The molecular mechanisms responsible for the progression and resistance to naCHT in sporadic or NF1-associated MPNST, are largely unknown. Biallelic *NF1* gene inactivation is not enough for progression toward MPNST, and additional genetic alterations are necessary (Sohier et al. 2017). They are thought to involve the factors regulating the tumor cell-cycle, apoptosis and invasion (Levy et al. 2004). Recent publications have suggested that the cell-cycle pathway and its multiple protein components, including survivin, cyclin D1 and p53, are frequently altered in cancer. In addition, ECM proteins, OPN and FN, were shown to play important roles in cancers’ invasion, neoagiogenesis, metastasis and progression. The impact of these markers on the response to naCHT in childhood MPNST has not been evaluated so far.

In the present study, we found that all analyzed tumor markers were expressed in at least half of the samples of primary chemo-naive MPNST tumors. High expressions of survivin and OPN were detected most commonly, in 76 and 67% of cases, respectively. Many neoplasms, including carcinomas of breast, prostate, lung, colon, bladder, esophagus, as well as lymphomas, neuroblastomas and osteosarcomas, were previously shown to overexpress survivin (Tanaka et al. 2000; Xing et al. 2001; Kato et al. 2001). High expression of survivin was also reported in over 80% of RMS tumors, the most common subtype of pediatric STS. The exact role of this protein in pathogenesis and invasion of STS is not clear, however, survivin expression was suggested to predominate in more aggressive and invasive tumors (Fangusaro et al. 2005). Alaggio et al. reported that high expression of survivin mRNA correlated with shorter survival and more aggressive clinical behavior of adult MPNST (Alaggio et al. 2013).

Due to the rarity of STS, the data on the prognostic role of OPN in these tumors are limited. However, Bramwell et al. have shown that benign mesenchymal tumors, such as lipomas, borderline-malignant dermatofibrosarcomas and well-differentiated/myxoid liposarcomas do not express OPN, while other subtypes of malignant STS of histological grades 2 or 3, display strong expression of OPN in over 80% of samples (Bramwell et al. 2005). High tumor OPN expression was reported to correlate with higher grade, stage and shorter relapse-free survival and overall survival in adult STS (Bache et al. 2010). In pediatric cancers, the tumor expression of OPN was examined in leukemias, lymphomas, Langerhans cell histiocytosis, central nervous system (CNS) tumors, osteosarcoma and renal tumors (summarized by Karpinsky et al. 2017). To date, there has been no study on the prognostic role of this marker in children with MPNST or other STS subtypes.

Our study was the first to determine whether pre-treatment tumor expressions of survivin, p53, cyclin D1, OPN and FN might help to predict the response to naCHT in pediatric inoperable and/or metastatic MPNST. It appeared that all analyzed markers were more frequently expressed in poor responders to naCHT, than in good responders. Survivin was expressed in all, OPN, p53 and FN—in 81.8% each, and cyclin D1 in 72.7% of tumor samples obtained from patients, who responded poor to naCHT. In good responders, the tumor expressions of particular markers were less frequent (50, 20, 40, 20 and 50%, respectively). It was found in univariate analysis, that high tumor expressions of survivin, p53, and OPN correlated significantly with poor response to naCHT. The tumor expressions of cyclin D1 and of FN were also higher in poor responders, than in good responders, however, the differences between the groups were insignificant. Our findings suggest that overexpression of the markers involved in tumor cells apoptosis, cell-cycle regulation and cancer invasiveness, may contribute to the resistance to CHT in childhood MPNST.

The influence of survivin, an anti-apoptotic protein, on the response to CHT and RTX is uncertain. In head and neck cancer, higher tumor survivin expression was associated with a better response to RTX and longer survival. Accordingly, survivin silencing in cell lines led to decreased sensitivity to radiation (Farnebo et al. 2013). To the contrary, in the cancers of esophagus, breast and kidney, high IHC expression of survivin was correlated with a poor response to RTX (Jha et al. 2011). Additionally, inhibition of survivin was shown to sensitize tumor cells to different chemotherapeutic agents, including cisplatin (Zaffaroni and Daidone 2002). In our study, all poor responders were found to overexpress survivin while all five patients expressing negatively for survivin, responded well to naCHT (Table 3).

It has been suggested that treatment sensitivity of cancers is influenced by the altered function of p53 protein, resulting from the mutations of the *TP53* tumor suppressor gene (Shukla et al. 2013). In NF1-related MPNST, the loss or mutation in the *TP53* gene has been associated with an increased proliferative potential, formation of metastases and particularly poor prognosis (Upadhyaya et al. 1997). Brekke et al. have shown that patients with NF1-associated MPNST, in whom the accumulation of nuclear p53 was found, form a high-risk subgroup requiring adjuvant treatment, even when in complete remission (Brekke et al. 2009). In our study, 8 of 10 patients with low tumor p53 expression responded well to naCHT, while 9 of 11 children with high p53 immunoreactivity responded poor.

Our review of all publications on the role of OPN in cancers of children and young adults has shown that the monitoring of OPN protein level in serum and cerebral-spinal fluid of children with acute lyphoblastic anemia with CNS involvement and in patients with highly malignant brain tumors, reflected the tumor bulk and the response to CHT (Karpinsky et al. 2017). To date, there have been no publications on the impact of OPN expression on response to naCHT in patients with STS. In our series of children with MPNST, five of seven patients with low OPN expression responded well to naCHT.

Cyclin D1 has been frequently found deregulated in cancer and is regarded as a biomarker of invasive cancer phenotype. It has been also shown to influence the response to CHT and RTX (Shintani et al. 2001). Following exposure to environmental stress or DNA damage, the rapid degradation of cyclin D1 ensures rapid cell cycle arrest (Yang et al. 2006). Several therapeutic agents have been shown to induce cyclin D1 degradation (Feng et al. 2007). The cyclin D1 accumulation and overexpression has led to increased chemotherapeutic resistance and protection from apoptosis (Shintani et al. 2002). Patients with head and neck squamous cell carcinomas (HNSCC) with high cyclin D1 tumor expressions were less likely to respond to neo-adjuvant cisplatin-based CHT and survive, than patients with low expressions of the marker (58 vs. 85%; *p* < 0.001). It was concluded that tumor expression of cyclin D1 may serve as a predictive biomarker in selecting patients with HNSCC, who may benefit from naCHT (Feng et al. 2011). Several studies have also demonstrated abnormalities of both the p53 and the Rb1-cyclin D1 pathways in MPNST (Kourea et al. 1999).

The processes of the cell-cycle regulation, tumor cells’ proliferation and invasion, inhibition of apoptosis and promotion of neo-angiogenesis, mediated within tumor microenvironment by survivin, p53, cyclin D1, OPN and FN, are frequently interdependent. Moreover, these factors have been found to share mutual mechanisms and signaling pathways. Not surprisingly, we have found significant positive correlations between p53 and cyclin D1, OPN and p53 and cyclin D1 and FN. Understandably, poor response to naCHT was significantly more frequent in children with simultaneous high expression of p53 and cyclin D1 and OPN and p53 and also of survivin and cyclin D1, in comparison to patients not expressing any of these markers. Simultaneous high expressions of OPN, p53 and cyclin D1 were found significantly more frequently in poor responders, than no expression of these markers (p = 0.005). Generally, the more markers were expressed simultaneously in a tumor tissue, the worse was the response to naCHT. Patients expressing all five markers responded poor to CHT, while four of five patients with no markers or only one marker expressed—responded well to CHT. Interestingly, the tumor expression of FN, either alone, or together with other markers, did not correlate with the response to naCHT in children with MPNST. Several previous studies have confirmed the role of FN in the pathogenesis of RMS and the invasiveness of adults RMS FN has been also suggested to be essential for lung metastasis in STS (Ito et al. 2004). However, FN determination at diagnosis in chemo-naive tumor samples of pediatric MPNST did not add significant prognostic information.

## Conclusions

1. The response to naCHT in advanced pediatric MPNST is influenced by the NF1 status and initial tumor tissue expression of OPN, survivin, p53 and cyclin D1.
2. Patients with high tumor expression of either OPN, survivin or p53 and those with simultaneous high expression of ≥ 3 of the analyzed markers, responded significantly worse to naCHT, than patients, in whom expression of ≤ 2 markers were detected at diagnosis.
3. The role of FN in predicting response to naCHT—as a sole analyzed marker and together with others—was not confirmed.
4. Future studies in more numerous groups of patients are needed to confirm these preliminary results.
